# Supplementary material for: Characterizing the Genomic Profile in High-Grade Gliomas: From Tumor Core to Peritumoral Brain Zone, Passing through Glioma-Derived Tumorspheres
Source: Biology (Basel). 2021 Nov 9;10(11):1157. doi: 10.3390/biology10111157 (PMC8615186; doi:10.3390/biology10111157)
Supplement: Supplementary file 1 [file biology-10-01157-s001.zip › biology-1428670-supplementary.pdf]

SUPPLEMENTARY FILES

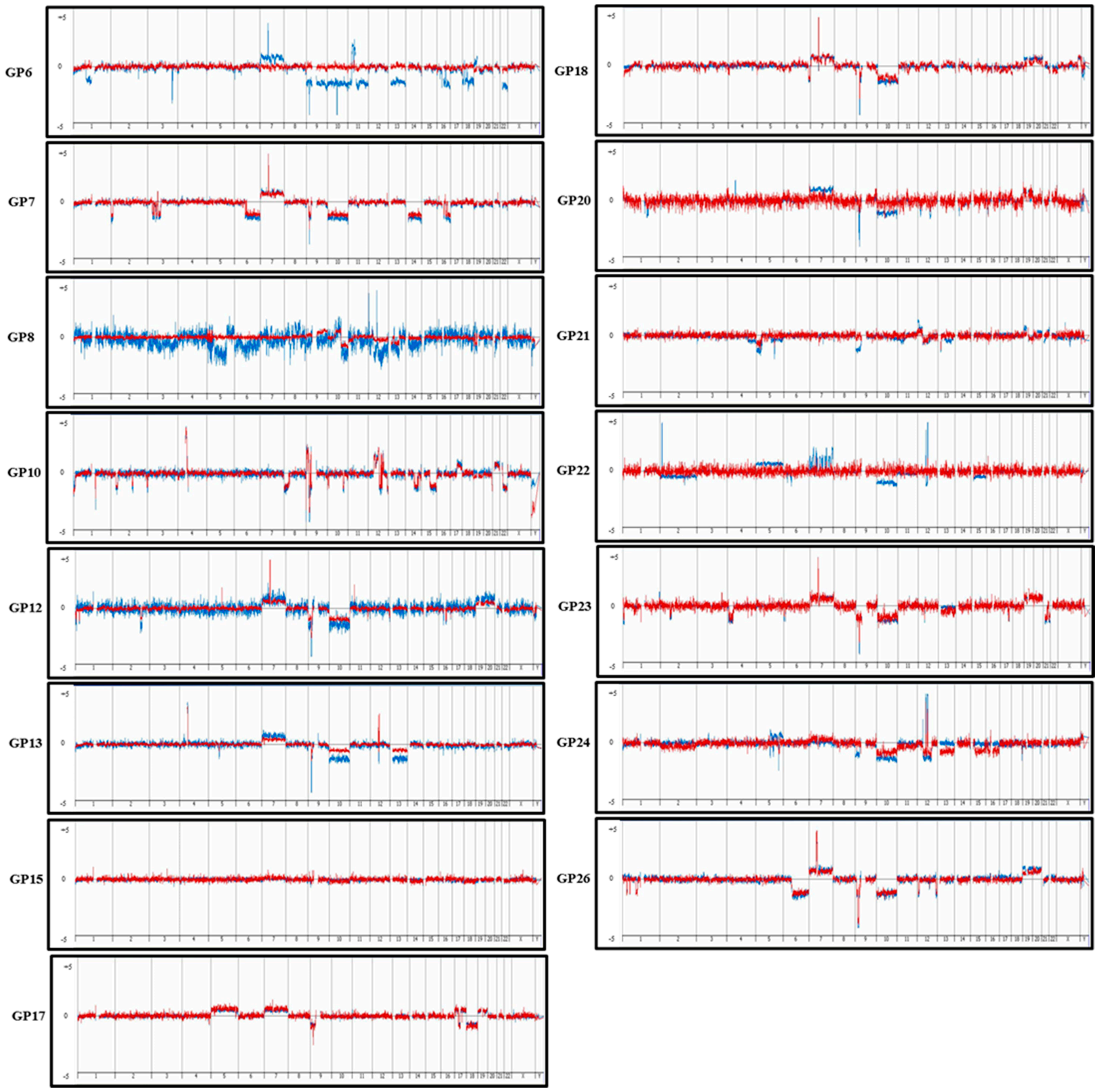

Figure S1. Genomic profiles of TCs and matched GSCs. Line plots show the genome comparison between TCs and matched GSCs, of a specific patient (GP). Red lines indicate biopies' genomic profiles while blue lines indicate stem cultures' ones; x-axis indicates the chromosomes; y-axis indicates the log2ratio values ranging from -5 to +5; trends beyond 0 as log2ratio value indicates gains, while trends below 0 as log2ratio indicates losses.

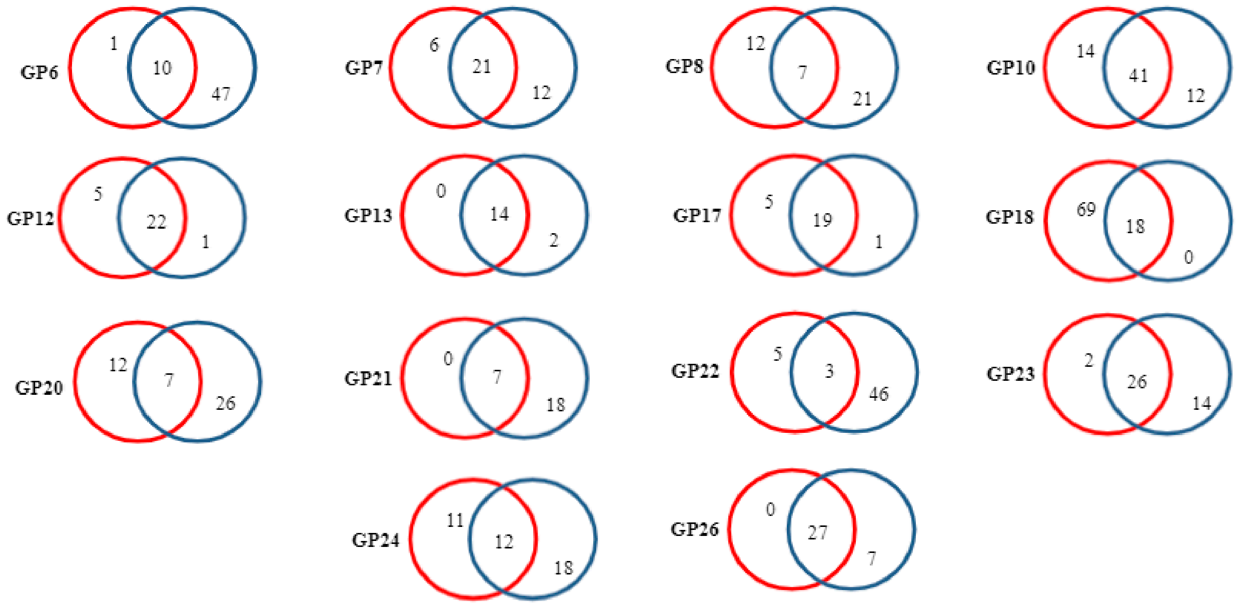

Figure S2. Shared CNAs TC -GSC. Number of overlapping shared CNAs between TC (red) and matched GSC culture (blue) genomic profiles, for each patient (GP).

Table S1. Shared CNAs TC-GSC. Summary of the shared CNAs between TC-GSC pairs for each patient (GP). For each imbalance is reported type of alteration (loss or gain), its locus , lenght (number of pairs of bases) and the percentage of mosaicism calculated.

Common CNA		GP6_TC		GP6_GSC	
LOSS	**3q27.1**	Mosaic 41%	**0,253 Mb**	Mosaic 39%	**1 Mb**
LOSS	**8q24.3**	Mosaic 34%	**3,4 Mb**	Mosaic 50%	**3,8 Mb**
LOSS	**10q26.3**	Mosaic 40%	**0,75 Mb**	No mosaic	**1,4 Mb**
LOSS	**11p15.5 - p15.4**	Mosaic 34%	**3,8 Mb**	No mosaic	**3,8 Mb**
LOSS	**16p13.3**	Mosaic 44%	**1,3 Mb**	Mosaic 57%	**1,1 Mb**
LOSS	**16q13**	Mosaic 39%	**0,413 Mb**	No mosaic	**1,5 Mb**
LOSS	**17q25.3**	Mosaic 45%	**1 Mb**	Mosaic 63%	**1 Mb**
LOSS	**22q11.21**	Mosaic 48%	**1,8 Mb**	No mosaic	**34 Mb**
LOSS	**Xp11.23**	Mosaic 34%	**0,833 Mb**	Mosaic 40%	**932 Mb**
LOSS	**Xq28**	Mosaic 33%	**1,3 Mb**	Mosaic 43%	**2 Mb**
**Common CNA**		**GP7_TC**		**GP7_GSC**	
LOSS	**1p33**	Mosaic 77%	**0,217 Mb**	No mosaic	**0,217 Mb**
LOSS	**2p25.3 -p24.3**	No mosaic	**12 Mb**	No mosaic	**12 Mb**
LOSS	**3p24.1 -p23**	Mosaic 62%	**0,881 Mb**	No mosaic	**0,881 Mb**
LOSS	**3p22.3 - p12.1**	Mosaic 38%	**54,2 Mb**	Mosaic 52%	**54 Mb**
GAIN	**3p14.1**	Mosaic 62%	**4,4 Mb**	Mosaic 65%	**4,4 Mb**
LOSS	**6q13 - q14.1**	Mosaic 71%	**8,5 Mb**	No mosaic	**5,4 Mb**
LOSS	**6q14.1**	Mosaic 71%	**8,5 Mb**	No mosaic	**0,509 Mb**
LOSS	**6q14.1 - q27**	Mosaic 75%	**88,3 Mb**	No mosaic	**88,3 Mb**
GAIN	**7p22.2 - p11.1**	No mosaic	**57,3 Mb**	No mosaic	**54,7 Mb**
GAIN	**7q11.21 - q36.3**	No mosaic	**96,4 Mb**	No mosaic	**96,2 Mb**
LOSS	**9p22.3 - p21.1**	Mosaic 77%	**15,4 Mb**	No mosaic	**15,8 Mb**
LOSS	**10p15.3 - p11.21**	Mosaic 78%	**38,4 Mb**	No mosaic	**38,4 Mb**
LOSS	**10q11.21 - q26.3**	Mosaic 79%	**93 Mb**	No mosaic	**93 Mb**
GAIN	**12q24.31**	No mosaic	**1,1 Mb**	Mosaic 76%	**1 Mb**
LOSS	**14q11.2 - q32.33**	Mosaic 78%	**87 Mb**	No mosaic	**87 Mb**
LOSS	**16q12.1**	Mosaic 76%	**2,8 Mb**	No mosaic	**2,8 Mb**
GAIN	**16q13 - q21**	No mosaic	**1,2 Mb**	Mosaic 74%	**1 Mb**
LOSS	**16q21 - q22.1**	Mosaic 79%	**8,6 Mb**	No mosaic	**8,6 Mb**
LOSS	**16q22.3 - q24.3**	No mosaic	**18 Mb**	No mosaic	**18 Mb**
GAIN	**17q25.1**	No mosaic	**0,317 Mb**	No mosaic	**0,3 Mb**

GAIN	**21q22.11**	No mosaic	**1,3 Mb**	No mosaic	**1,2 Mb**
**Common CNA**		**GP8_TC**		**GP8_GSC**	
LOSS	**5p14.2**	Mosaic 50%	**0,623 Mb**	No mosaic	**10 Mb**
LOSS	**10q23.2 - q26.3**	Mosaic 56%	**45,6 Mb**	No mosaic	**45,6 Mb**
LOSS	**13q12.11 - q12.13**	Mosaic 44%	**6,1 Mb**	Mosaic 66%	**55 Mb**
LOSS	**19p13.3 - 19p13.2**	Mosaic 39%	**7,2 Mb**	No mosaic	**2,6 Mb**
GAIN	**19p13.2**	Mosaic 30%	**4,2 Mb**	No mosaic	**4 Mb**
LOSS	**19p13.11 - p12**	Mosaic 40%	**8,9 Mb**	No mosaic	**1,9 Mb**
LOSS	**21q11.2**	Mosaic 51%	**0,599 Mb**	Mosaic 59%	**12,1 Mb**
**Common CNA**		**GP10_TC**		**GP10_GSC**	
LOSS	**1p36.33 - p36.22**	No mosaic	**13 Mb**	No mosaic	**8 Mb**
LOSS	**1q21.1**	No mosaic	**0,112 Mb**	No mosaic	**0,112 Mb**
LOSS	**2p22.3 - p21**	No mosaic	**11,6 Mb**	No mosaic	**11,6 Mb**
LOSS	**2q22.2 - q23.1**	Mosaic 72%	**5,3 Mb**	No mosaic	**5,7 Mb**
LOSS	**2q37.1**	No mosaic	**0,336 Mb**	No mosaic	**0,319 Mb**
LOSS	**2q37.3**	No mosaic	**0,796 Mb**	No mosaic	**0,83 Mb**
LOSS	**3p26.3**	No mosaic	**2,9 Mb**	No mosaic	**2,9 Mb**
GAIN	**4q11 - q13.1**	No mosaic	**8,3 Mb**	No mosaic	**8,2 Mb**
GAIN	**4q12**	No mosaic	**3,3 Mb**	No mosaic	**3,3 Mb**
GAIN	**4q13.1**	No mosaic	**0,736 Mb**	No mosaic	**0,736 Mb**
GAIN	**4q13.1**	No mosaic	**1,4 Mb**	No mosaic	**1,2 Mb**
LOSS	**4q13.2**	No mosaic	**1,5 Mb**	No mosaic	**1,4 Mb**
LOSS	**8p23.3 - p12**	No mosaic	**31,6 Mb**	No mosaic	**31,7 Mb**
LOSS	**8p12**	Mosaic 70%	**1,2 Mb**	No mosaic	**0,725 Mb**
LOSS	**8p11.23**	Mosaic 75%	**35,5 Mb**	No mosaic	**0,589 Mb**
LOSS	**9p24.3**	No mosaic	**0,41 Mb**	No mosaic	**0,41 Mb**
GAIN	**9p24.2 - p21.3**	No mosaic	**16,6 Mb**	No mosaic	**16,6 Mb**
LOSS	**9p21.3**	No mosaic	**5 Mb**	No mosaic	**5 Mb**
GAIN	**9p21.2**	No mosaic	**0,574 Mb**	No mosaic	**0,526 Mb**
LOSS	**9p21.2 - p21.1**	Mosaic 79%	**3,8 Mb**	No mosaic	**4,6 Mb**
GAIN	**9p21.1 - p13.3**	No mosaic	**2 Mb**	No mosaic	**2 Mb**
LOSS	**9p13.3 - p13.1**	No mosaic	**4,8 Mb**	No mosaic	**4,8 Mb**
LOSS	**10p15.3**	No mosaic	**2,5 Mb**	No mosaic	**2,5 Mb**
LOSS	**10p14**	Mosaic 72%	**1,6 Mb**	No mosaic	**1,6 Mb**
LOSS	**10q24.32 - q25.1**	No mosaic	**5,5 Mb**	No mosaic	**5,5 Mb**
GAIN	**12q12 - q21.1**	No mosaic	**35,2 Mb**	No mosaic	**35,2 Mb**
LOSS	**12q21.1 - q21.31**	Mosaic 69%	**11,3 Mb**	No mosaic	**11,3 Mb**
GAIN	**12q21.2**	No mosaic	**0,412 Mb**	No mosaic	**0,412 Mb**
LOSS	**12q22**	No mosaic	**2,6 Mb**	No mosaic	**2,7 Mb**
GAIN	**12q22 - q23.3**	No mosaic	**8 Mb**	No mosaic	**3,9 Mb**
LOSS	**12q24.32 - q24.33**	No mosaic	**6,5 Mb**	No mosaic	**6,5 Mb**
LOSS	**14q23.1 - q31.3**	Mosaic 73%	**28,8 Mb**	No mosaic	**28,8 Mb**
LOSS	**14q32.2**	Mosaic 77%	**1,8 Mb**	No mosaic	**1,8 Mb**
LOSS	**15q22.1 - q26.3**	Mosaic 78%	**43,6 Mb**	No mosaic	**44 Mb**
GAIN	**17q21.32 - q25.3**	Mosaic 72%	**34,3 Mb**	No mosaic	**34, 6 Mb**
LOSS	**20q13.33**	No mosaic	**2,4 Mb**	No mosaic	**2,6 Mb**
GAIN	**21q11.2 - q22.3**	No mosaic	**32,8 Mb**	No mosaic	**33,3 Mb**
GAIN	**22q11.1 - q11.21**	Mosaic 50%	**4,3 Mb**	No mosaic	**4 Mb**
LOSS	**22q11.21 - q13.33**	No mosaic	**29,3 Mb**	No mosaic	**29,2 Mb**
LOSS	**Yp11.31 - p11.2**	No mosaic	**7,8 Mb**	Mosaic 55%	**8,7 Mb**
LOSS	**Yq11.21 - q11.23**	No mosaic	**14,6 Mb**	Mosaic 67%	**45,1 Mb**
**Common CNA**		**GP12_TC**		**GP12_GSC**	
LOSS	**1p36.23 - p36.22**	No mosaic	**1,3 Mb**	No mosaic	**1,5 Mb**
LOSS	**1p36.11 - p35.3**	Mosaic 70%	**2,2 Mb**	Mosaic 79%	**2,3 Mb**
LOSS	**2q32.1 - q32.3**	Mosaic 65%	**10,4 Mb**	No mosaic	**10,3 Mb**
LOSS	**3q29**	Mosaic 67%	**0,41 Mb**	No mosaic	**0,196 Mb**
GAIN	**5q14.3**	No mosaic	**0,075 Mb**	No mosaic	**0,075 Mb**
GAIN	**7p22.3 - p11.1**	Mosaic 77%	**57,3 Mb**	No mosaic	**54,4 Mb**
GAIN	**7p11.2**	No mosaic	**2 Mb**	No mosaic	**2,1 Mb**
GAIN	**7q11.21 - q36.3**	Mosaic 71%	**96,6 Mb**	No mosaic	**96 Mb**
LOSS	**9p24.3 - p22.1**	Mosaic 66%	**18,8 Mb**	No mosaic	**19 Mb**

LOSS	**9p21.3**	No mosaic	**5 Mb**	No mosaic	**5 Mb**
LOSS	**9p21.2 - p21.1**	Mosaic 52%	**1,9 Mb**	No mosaic	**4 Mb**
LOSS	**10p15.3 - p11.21**	Mosaic 68%	**38,4 Mb**	No mosaic	**38,4 Mb**
LOSS	**10q11.21 - q26.3**	Mosaic 70%	**93 Mb**	No mosaic	**93 Mb**
LOSS	**11q23.3**	No mosaic	**0,934 Mb**	No mosaic	**1 Mb**
LOSS	**12q24.33**	Mosaic 68%	**0,641 Mb**	No mosaic	**0,853 Mb**
LOSS	**16q12.1**	Mosaic 79%	**1,3 Mb**	No mosaic	**0,940 Mb**
LOSS	**16q12.2**	Mosaic 79%	**0,565 Mb**	No mosaic	**0,394 Mb**
LOSS	**16q23.2**	Mosaic 73%	**0,482 Mb**	No mosaic	**0,543 Mb**
GAIN	**19p13.3 - p12**	Mosaic 45%	**24 Mb**	No mosaic	**23,7 Mb**
GAIN	**19 q12 - q13.43**	Mosaic 50%	**30,5 Mb**	No mosaic	**30 Mb**
GAIN	**20p13 - p11.1**	Mosaic 68%	**26 Mb**	No mosaic	**26 Mb**
GAIN	**20q11.21 - q13.33**	Mosaic 57%	**33 Mb**	No mosaic	**33 Mb**
	**Common CNA**	**GP13_TC**		**GP13_GSC**	
GAIN	**4q12**	No mosaic	**3,4 Mb**	No mosaic	**3,4 Mb**
LOSS	**5q13.2**	Mosaic 40%	**2,7 Mb**	No mosaic	**2,3 Mb**
GAIN	**7p22.2 - p11.1**	Mosaic 44%	**57 Mb**	No mosaic	**55 Mb**
GAIN	**7q11.21 - q36.3**	Mosaic 45%	**96,5 Mb**	No mosaic	**96,5 Mb**
LOSS	**9p21.3 - p21.1**	Mosaic 60%	**7,4 Mb**	No mosaic	**2,3 Mb**
LOSS	**9p21.3**	Mosaic 89%	**1,9 Mb**	No mosaic	**4,6 Mb**
LOSS	**10p15.3 - p11.21**	Mosaic 43%	**38,4 Mb**	No mosaic	**38,4 Mb**
LOSS	**10q11.21 - q26.3**	Mosaic 45%	**93 Mb**	No mosaic	**93 Mb**
GAIN	**12q13.3**	No mosaic	**0,120 Mb**	No mosaic	**0,149 Mb**
LOSS	**12q13.3 - q14.1**	Mosaic 45%	**0,152 Mb**	No mosaic	**0,152 Mb**
GAIN	**12q14.1**	No mosaic	**0,044 Mb**	No mosaic	**0,12 Mb**
GAIN	**12q14.1**	No mosaic	**0,044 Mb**	No mosaic	**0,035 Mb**
LOSS	**12q14.1**	Mosaic 45%	**3,2 Mb**	No mosaic	**3,3 Mb**
LOSS	**13q11 - q34**	Mosaic 42%	**96 Mb**	No mosaic	**96 Mb**
	**Common CNA**	**GP15_TC**		**GP15_GSC**	
	NO COMMON CNA	-	-	-	-
	**Common CNA**	**GP17_TC**		**GP17_GSC**	
LOSS	**1p36.12 - p36.11**	No mosaic	**0,179 Mb**	Mosaic 47%	**0,341 Mb**
LOSS	**4q34.3**	Mosaic 53%	**1,4 Mb**	Mosaic 53%	**1,4 Mb**
GAIN	**5p15.33 - p11**	Mosaic 74%	**44 Mb**	Mosaic 58%	**46 Mb**
GAIN	**5q11.1 - q35.3**	Mosaic 76%	**131 Mb**	Mosaic 58%	**131 Mb**
GAIN	**7p22.3 - p11.2**	Mosaic 75%	**56,5 Mb**	Mosaic 56%	**57 Mb**
GAIN	**7q11.21 - q36.3**	Mosaic 73%	**96 Mb**	Mosaic 56%	**96 Mb**
LOSS	**9p24.3 - p13.3**	Mosaic 75%	**33,7 Mb**	Mosaic 59%	**33,7 Mb**
GAIN	**17p13.3 - p11.2**	Mosaic 60%	**22 Mb**	Mosaic 47%	**21,3 Mb**
LOSS	**17p13.1**	Mosaic 65%	**0,343 Mb**	Mosaic 58%	**0,378 Mb**
GAIN	**17q11.1 - q11.2**	Mosaic 79%	**4 Mb**	Mosaic 52%	**4 Mb**
LOSS	**17q11.2**	Mosaic 71%	**0,844 Mb**	Mosaic 55%	**0,844 Mb**
GAIN	**17q11.2 - q12**	Mosaic 61%	**2,1 Mb**	Mosaic 48%	**2,1 Mb**
LOSS	**17q12 - q21.31**	Mosaic 70%	**10,3 Mb**	Mosaic 54%	**10,4 Mb**
GAIN	**17q21.31 - q25.3**	Mosaic 64%	**38,3 Mb**	Mosaic 48%	**38,3 Mb**
LOSS	**17q22**	Mosaic 78%	**0,58 Mb**	Mosaic 42%	**717 Mb**
LOSS	**18p11.32 - p11.21**	Mosaic 69%	**14,6 Mb**	Mosaic 52%	**14,6 Mb**
LOSS	**18q11.1 - q23**	Mosaic 65%	**59,3 Mb**	Mosaic 53%	**59,3 Mb**
GAIN	**19p13.3 - p12**	Mosaic 52%	**23,8 Mb**	Mosaic 44%	**23,7 Mb**
GAIN	**19q12 - q13.43**	Mosaic 54%	**30,6 Mb**	Mosaic 44%	**30,6 Mb**
	**Common CNA**	**GP18_TC**		**GP18_GSC**	
LOSS	**1p34.3**	No mosaic	**0,743 Mb**	Mosaic, 76%	**0,786 Mb**
LOSS	**6q25.3 - q27**	Mosaic, 79%	**10,4 Mb**	No mosaic	**10,6 Mb**
GAIN	**7p22.1 - p11.2**	Mosaic, 76%	**50 Mb**	No mosaic	**55,8 Mb**
GAIN	**7q11.21 - q36.3**	Mosaic, 69%	**93,6 Mb**	No mosaic	**96,7 Mb**
LOSS	**9p21.3**	No mosaic	**2,4 Mb**	No mosaic	**2,4 Mb**
LOSS	**9p21.3 - p13.3**	Mosaic, 50%	**11 Mb**	No mosaic	**8,7 Mb**
LOSS	**10p15.3 - p11.21**	Mosaic, 79%	**38,4 Mb**	No mosaic	**38,3 Mb**
LOSS	**10q11.21 - q26.3**	No mosaic	**93 Mb**	No mosaic	**93 Mb**
LOSS	**19p13.3 - p13.2**	Mosaic, 51%	**0,416 Mb**	Mosaic, 73%	**23,9 Mb**
LOSS	**19p13.2**	Mosaic, 47%	**1 Mb**	Mosaic, 73%	**23,9 Mb**

LOSS	**19p13.2 - p13.13**	Mosaic, 46%	**1 Mb**	Mosaic, 73%	**23,9 Mb**
LOSS	**19p13.11 - p12**	Mosaic, 61%	**4,5 Mb**	Mosaic, 73%	**23,9 Mb**
LOSS	**19q13.12**	Mosaic, 69%	**1,4 Mb**	Mosaic, 77%	**30,8 Mb**
LOSS	**19q13.42 - q13.43**	Mosaic, 51%	**2, 5 Mb**	Mosaic, 77%	**30,8 Mb**
LOSS	**20p13 - p11.1**	Mosaic, 56%	**26,1 Mb**	No mosaic	**26 Mb**
LOSS	**20q11.21 - q13.33**	Mosaic, 34%	**30 Mb**	Mosaic, 77%	**33 Mb**
LOSS	**Xq26.3 - q28**	Mosaic, 76%	**17,6 Mb**	No mosaic	**20,8 Mb**
LOSS	**Xq28**	Mosaic, 79%	**1,3 Mb**	No mosaic	**20,8 Mb**
**Common CNA**		**GP20_TC**		**GP20_GSC**	
GAIN	**7p22.3 - p11.2**	Mosaic 35%	**55,8 Mb**	No mosaic	**57,3 Mb**
GAIN	**7q11.21 - q36.3**	Mosaic 25%	**96,3 Mb**	No mosaic	**96,6 Mb**
GAIN	**9q34.3**	Mosaic 70%	**12,7 Mb**	No mosaic	**0,255 Mb**
LOSS	**10q21.1**	No mosaic	**1,2 Mb**	Mosaic 79%	**93 Mb**
GAIN	**16q24.3**	Mosaic 67%	**5,6 Mb**	No mosaic	**37,9 Mb**
GAIN	**19p13.3 - p13.11**	No mosaic	**19,4 Mb**	No mosaic	**23,8 Mb**
GAIN	**19q13.11 - q13.42**	No mosaic	**23,7 Mb**	Mosaic 77%	**30,8 Mb**
**Common CNA**		**GP21_TC**		**GP21_GSC**	
LOSS	**5p15.33 - p13.3**	Mosaic 56%	**32 Mb**	No mosaic	**31,4 Mb**
LOSS	**5p13.2**	Mosaic 72%	**0,729 Mb**	No mosaic	**0,996 Mb**
LOSS	**5q23.1**	Mosaic 71%	**2,3 Mb**	No mosaic	**2,6 Mb**
GAIN	**12p13.33 - p11.21**	Mosaic 35%	**32 Mb**	No mosaic	**32 Mb**
LOSS	**12q12 - q15**	Mosaic 37%	**32,3 Mb**	Mosaic 39%	**32,3 Mb**
LOSS	**13q12.11**	Mosaic 79%	**2,9 Mb**	No mosaic	**3,3 Mb**
GAIN	**Xp11.22**	No mosaic	**0,646 Mb**	No mosaic	**0,823 Mb**
**Common CNA**		**GP22_TC**		**GP22_GSC**	
GAIN	**5p13.2**	No mosaic	**0,088 Mb**	Mosaic, 75%	**45,9 Mb**
GAIN	**11p12 - p11.2**	Mosaic, 46%	**0,567 Mb**	Mosaic, 61%	**0,567 Mb**
GAIN	**12q15**	No mosaic	**1,6 Mb**	No mosaic	**1,9 Mb**
**Common CNA**		**GP23_TC**		**GP23_GSC**	
LOSS	**1p36.32 - p36.23**	Mosaic 73%	**5 Mb**	No Mosaic	**5,3 Mb**
GAIN	**1p36.13 - p36.12**	No Mosaic	**1 Mb**	No Mosaic	**0,92 Mb**
GAIN	**1q21.3 - q22**	No Mosaic	**1 Mb**	No Mosaic	**0,986 Mb**
GAIN	**1q43 - q44**	Mosaic 62%	**3,3 Mb**	No Mosaic	**3,6 Mb**
LOSS	**2p14 - p13.3**	Mosaic 53%	**8,1 Mb**	Mosaic 68%	**8,7 Mb**
LOSS	**4p16.1 - p15.33**	Mosaic 63%	**129 Mb**	No Mosaic	**129 Mb**
LOSS	**4p15.32 - p14**	Mosaic 73%	**24,6 Mb**	No Mosaic	**24,6 Mb**
GAIN	**7p22.3 - p12.1**	No Mosaic	**53,6 Mb**	No Mosaic	**57,3 Mb**
GAIN	**7p11.2**	No Mosaic	**1 Mb**	No Mosaic	**1 Mb**
GAIN	**7q11.21 - q36.3**	Mosaic 76%	**94,2 Mb**	No Mosaic	**96,4 Mb**
LOSS	**9p24.3 - p13.1**	No Mosaic	**38,8 Mb**	No Mosaic	**38,8 Mb**
LOSS	**9p21.3**	No Mosaic	**1,8 Mb**	No Mosaic	**1,8 Mb**
LOSS	**10p15.3 - p11.21**	Mosaic 77%	**38 Mb**	No Mosaic	**38 Mb**
LOSS	**10q11.21 - q26.3**	Mosaic 72%	**92,5 Mb**	No Mosaic	**93 Mb**
LOSS	**12q12 - q13.11**	No Mosaic	**0,673 Mb**	No Mosaic	**0,858 Mb**
GAIN	**12q13.12**	No Mosaic	**0,452 Mb**	No Mosaic	**0,930 Mb**
GAIN	**15q22.31**	No Mosaic	**0,758 Mb**	No Mosaic	**0,758 Mb**
GAIN	**15q26.1**	No Mosaic	**0,654 Mb**	No Mosaic	**0,654 Mb**
LOSS	**16p13.3**	No Mosaic	**0,433 Mb**	No Mosaic	**0,433 Mb**
GAIN	**16q13**	No Mosaic	**0,803 Mb**	No Mosaic	**0,938 Mb**
LOSS	**17q22 - q23.2**	Mosaic 63%	**3,9 Mb**	Mosaic 62%	**3,9 Mb**
GAIN	**19p13.3 - p13.11**	No Mosaic	**19,3 Mb**	No Mosaic	**23,7 Mb**
GAIN	**19q12 - q13.43**	No Mosaic	**30,6 Mb**	No Mosaic	**30,5 Mb**
GAIN	**20p13 - p11.1**	No Mosaic	**25,4 Mb**	No Mosaic	**25,9 Mb**
GAIN	**20q11.21 - q13.33**	No Mosaic	**33 Mb**	No Mosaic	**33 Mb**
LOSS	**21q11.2 - q22.3**	Mosaic 67%	**7,7 Mb**	No Mosaic	**32,7 Mb**
**Common CNA**		**GP24_TC**		**GP24_GSC**	
GAIN	**7p14.1**	Mosaic 43%	**55 Mb**	No mosaic	**55,5 Mb**
LOSS	**10p15.3 - p11.21**	Mosaic 68%	**38,4 Mb**	No mosaic	**38,4 Mb**
LOSS	**10q11.21 - q26.3**	Mosaic 62%	**93 Mb**	No mosaic	**93 Mb**
LOSS	**12q12 - q13.3**	Mosaic 61%	**18 Mb**	No mosaic	**17,5 Mb**
GAIN	**12q13.3 - q14.1**	No mosaic	**0,197 Mb**	No mosaic	**1,5 Mb**

LOSS	**12q14.1 - q14.3**	Mosaic 65%	**7,6 Mb**	No mosaic	**7,6 Mb**
GAIN	**12q15**	No mosaic	**1,6 Mb**	No mosaic	**0,144 Mb**
GAIN	**12q21.1**	No mosaic	**0,114 Mb**	No mosaic	**5 Mb**
LOSS	**12q21.1 - q23.1**	Mosaic 67%	**24,6 Mb**	No mosaic	**24,2 Mb**
LOSS	**18q23**	Mosaic 60%	**2 Mb**	Mosaic 75%	**4 Mb**
GAIN	**Yp11.32 - p11.2**	Mosaic 55%	**7,8 Mb**	Mosaic 47%	**7,8 Mb**
GAIN	**Yq11.21 - q12**	Mosaic 50%	**45 Mb**	Mosaic 48%	**44,9 Mb**
**Common CNA**		**GP26_TC**		**GP26_GSC**	
LOSS	**1p35.3 - p35.1**	No mosaic	**3,9 Mb**	Mosaic 75%	**3,9 Mb**
LOSS	**1p33**	Mosaic 75%	**0,437 Mb**	No mosaic	**0,437 Mb**
LOSS	**1p33 - p32.3**	Mosaic 75%	**1,6 Mb**	No mosaic	**1,6 Mb**
GAIN	**4p15.2**	No mosaic	**0,377 Mb**	No mosaic	**0,377 Mb**
LOSS	**6p11.2 - p11.1**	Mosaic 63%	**1,3 Mb**	Mosaic 71%	**1,3 Mb**
LOSS	**6q11.1 - q27**	No mosaic	**108,7 Mb**	No mosaic	**108,7 Mb**
GAIN	**7p22.3 - p11.2**	No mosaic	**55,6 Mb**	No mosaic	**56,6 Mb**
GAIN	**7p12.2**	No mosaic	**0,724 Mb**	No mosaic	**0,724 Mb**
GAIN	**7p12.1**	No mosaic	**2 Mb**	No mosaic	**2 Mb**
GAIN	**7p11.2**	No mosaic	**1,3 Mb**	No mosaic	**1,3 Mb**
GAIN	**7q11.21 - q36.3**	No mosaic	**96,5 Mb**	No mosaic	**96,5 Mb**
LOSS	**9p23 - p22.1**	No mosaic	**7,9 Mb**	No mosaic	**7,9 Mb**
LOSS	**9p22.1 - p21.2**	No mosaic	**6,8 Mb**	No mosaic	**6,7 Mb**
LOSS	**9p22.1 - p21.3**	No mosaic	**6,8 Mb**	No mosaic	**0,641 Mb**
LOSS	**9p21.2 - p13.3**	Mosaic 78%	**6,5 Mb**	No mosaic	**6,5 Mb**
LOSS	**10p15.3 - p11.21**	No mosaic	**38,5 Mb**	No mosaic	**38,5 Mb**
LOSS	**10q11.21 - q26.3**	No mosaic	**93 Mb**	No mosaic	**93 Mb**
LOSS	**10q21.1**	No mosaic	**0,84 Mb**	No mosaic	**93 Mb**
LOSS	**10q23.1**	No mosaic	**0,184 Mb**	No mosaic	**93 Mb**
GAIN	**11q13.1**	No mosaic	**0,123 Mb**	No mosaic	**0,123 Mb**
LOSS	**12p13.31 - p12.3**	No mosaic	**6 Mb**	No mosaic	**6 Mb**
LOSS	**12q21.31**	No mosaic	**0,238 Mb**	No mosaic	**0,238 Mb**
LOSS	**12q24.31 - q24.33**	No mosaic	**6,7 Mb**	No mosaic	**6,7 Mb**
GAIN	**19p13.3 - p12**	Mosaic 65%	**23,6 Mb**	No mosaic	**23,6 Mb**
GAIN	**19q12 - q13.43**	Mosaic 70%	**30,8 Mb**	No mosaic	**30,8 Mb**
GAIN	**20p13 - p11.1**	No mosaic	**25,9 Mb**	No mosaic	**25,9 Mb**
GAIN	**20q11.21 - q13.33**	No mosaic	**33 Mb**	No mosaic	**33 Mb**

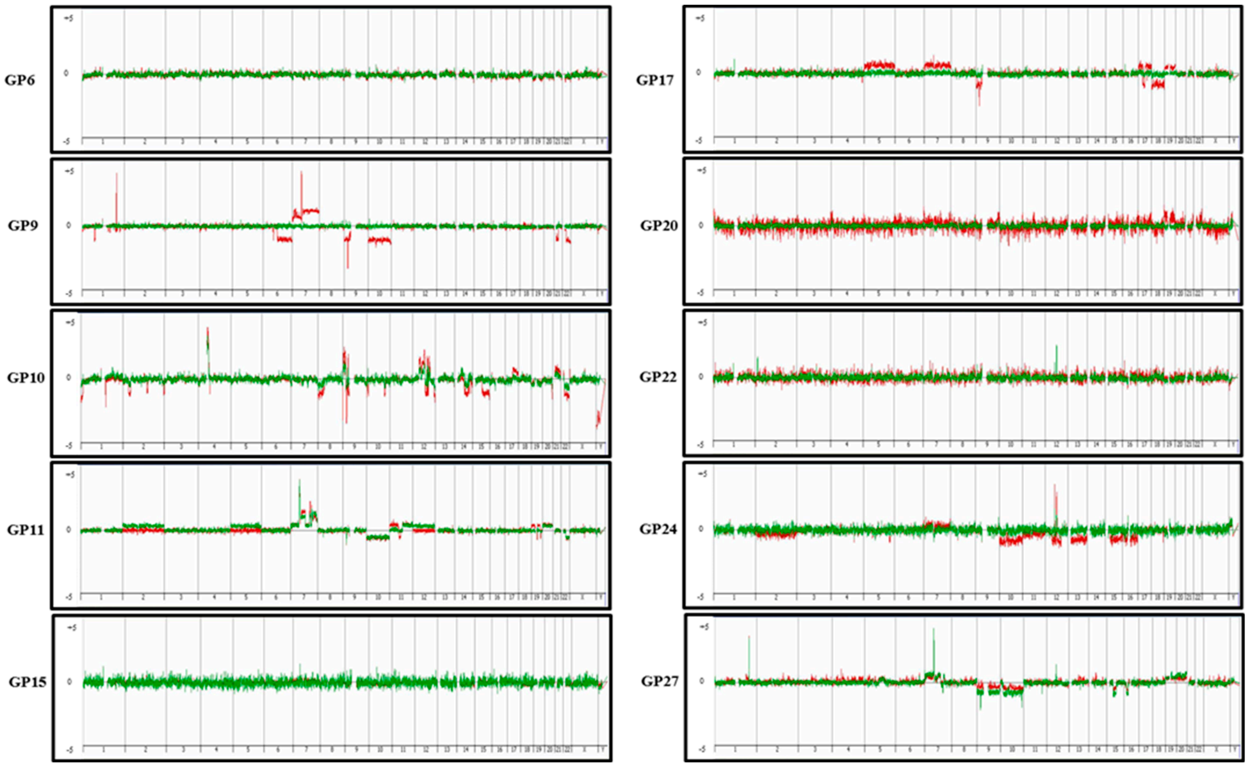

Figure S3. Genomic profiles of TCs and matched PBZs. Line plots show the genome comparison between TCs and PBZs, of a specific patient (GP). Red lines indicate tumor core genomic profiles while green lines indicate peritumoral brain zone ones; x-axis indicates the chromosomes; y-axis indicates the log2ratio values ranging from -5 to +5; trends beyond 0 as log2ratio value indicates gains, while trends below 0 as log2ratio indicates losses.

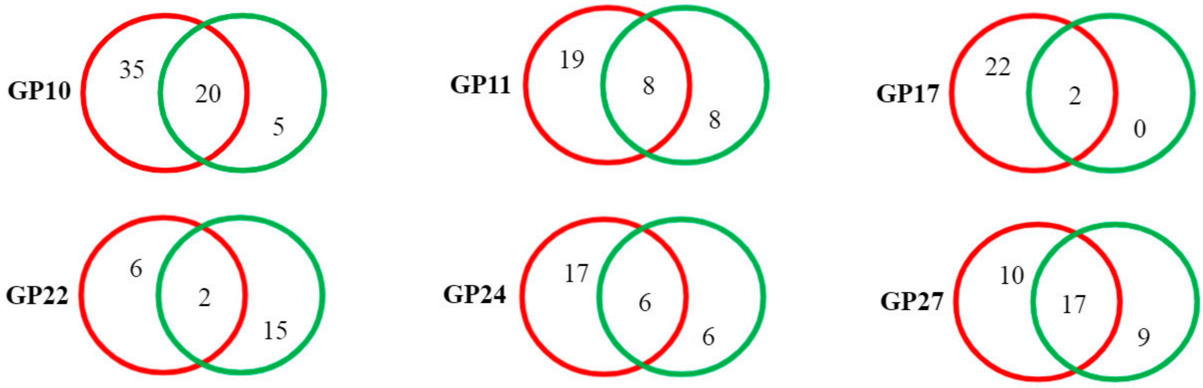

Figure S4. Shared CNAs TC-PBZ. Overlapping shared CNAs between TC (red) and matched PBZ (green) genomic profiles, for each patient (GP).

Table S2. Shared CNAs TC-PBZ. Summary of the shared CNAs between TC-PBZ pairs for each patient. For each imbalance is reported type of alteration (loss or gain), its locus , length (number of pairs of bases) and the percentage of mosaicism calculated.

Common CNA		GP6_TC		GP6_PBZ	
NO COMMON CNA		-	-	-	-
**Common CNA**		**GP9_TC**		**GP9_PBZ**	
NO COMMON CNA		-	-	-	-
**Common CNA**		**GP10_TC**		**GP10_PBZ**	
LOSS	**2p23 - p21**	Mosaic 79%	**11,7 Mb**	Mosaic 33%	**11,2 Mb**
GAIN	**4q12 - q13.1**	No mosaic	**8,3 Mb**	No mosaic	**8,1 Mb**
LOSS	**8p23.3 - p12**	No mosaic	**31,7 Mb**	Mosaic 38%	**31,7 Mb**
LOSS	**8p12**	Mosaic 69%	**1,2 Mb**	Mosaic 34%	**31,7 Mb**
LOSS	**8p11.23**	Mosaic 75%	**0,635 Mb**	Mosaic 34%	**2,3 Mb**
GAIN	**9p24.2 - p22.1**	No mosaic	**16,6 Mb**	No mosaic	**16,1 Mb**
LOSS	**9p21.3 - p21.2**	No mosaic	**1,3 Mb**	Mosaic 51%	**6,4 Mb**
GAIN	**9p21.1 - p13.3**	No mosaic	**2 Mb**	No mosaic	**2,5 Mb**
LOSS	**9p13.3 - p13.1**	No mosaic	**4,8 Mb**	Mosaic 39%	**4,2 Mb**
LOSS	**10p15.3**	No mosaic	**2,5 Mb**	Mosaic 38%	**4,1 Mb**
LOSS	**10q24.32 - q25.1**	No mosaic	**5,5 Mb**	Mosaic 46%	**4,2 Mb**
GAIN	**12q12 - q21.1**	No mosaic	**35,2 Mb**	No mosaic	**35,3 Mb**
GAIN	**12q21.31 - q21.32**	No mosaic	**1,4 Mb**	No mosaic	**1,6 Mb**
GAIN	**12q21.33 - q22**	No mosaic	**2,3 Mb**	No mosaic	**2,5 Mb**
LOSS	**12q22**	No mosaic	**2,6 Mb**	Mosaic 40%	**2,3 Mb**
GAIN	**12q22- q23.3**	No mosaic	**8 Mb**	Mosaic 47%	**7,9 Mb**
LOSS	**12q24.32 - q24.33**	No mosaic	**6,5 Mb**	Mosaic 50%	**6,4 Mb**
LOSS	**14q23.1 - q31.1**	Mosaic 73%	**28,8 Mb**	Mosaic 35%	**21,3 Mb**
LOSS	**20q13.33**	No mosaic	**2,5 Mb**	Mosaic 53%	**2,4 Mb**
LOSS	**22q11.21 - q13.33**	No mosaic	**29,3 Mb**	Mosaic 48%	**29,8 Mb**
**Common CNA**		**GP11_TC**		**GP11_PBZ**	
GAIN	**7p22.3 - p12.2**	Mosaic 50%	**50,5 Mb**	Mosaic 44%	**50,3 Mb**
GAIN	**7p12.2 - p11.2**	No mosaic	**4,7 Mb**	No mosaic	**4,8 Mb**
GAIN	**7p11.2 - p11.1**	No mosaic	**2 Mb**	No mosaic	**1,9 Mb**
GAIN	**7q11.21 - q36.3**	No mosaic	**96,6 Mb**	No mosaic	**96,6 Mb**
LOSS	**9p21.3**	No mosaic	**0,359 Mb**	Mosaic 74%	**0,96 Mb**
LOSS	**10p15.3 - p11.21**	Mosaic 48%	**38 Mb**	Mosaic 45%	**38 Mb**
LOSS	**10q11.21 - q26.3**	Mosaic 46%	**93 Mb**	Mosaic 44%	**93 Mb**
LOSS	**22q12.2 - q13.33**	Mosaic 48%	**20 Mb**	Mosaic 46%	**20 Mb**
**Common CNA**		**GP15_TC**		**GP15_PBZ**	
NO COMMON CNA		-	-	-	-
**Common CNA**		**GP17_TC**		**GP17_PBZ**	
GAIN	**1p31.1**	No mosaic	**0,134 Mb**	No mosaic	**0,134 Mb**
GAIN	**16q13**	No mosaic	**0,105 Mb**	No mosaic	**0,105 Mb**
**Common CNA**		**GP20_TC**		**GP20_PBZ**	
NO COMMON CNA		-	-	-	-
**Common CNA**		**GP22_TC**		**GP22_PBZ**	
GAIN	**5p13.2**	No mosaic	**0,088 Mb**	No mosaic	**0,015 Mb**
GAIN	**12q15**	No mosaic	**1,6 Mb**	No mosaic	**1,9 Mb**
**Common CNA**		**GP24_TC**		**GP24_PBZ**	
LOSS	**12q13.13**	Mosaic 61%	**18,1 Mb**	Mosaic 59%	**1,1 Mb**

GAIN	**12q13.3 - q14.1**	No mosaic	**0,365 Mb**	No mosaic	**0,36 Mb**
GAIN	**12q14.1**	No mosaic	**0,198 Mb**	No mosaic	**0,095 Mb**
GAIN	**12q15**	No mosaic	**1,6 Mb**	No mosaic	**0,776 Mb**
GAIN	**Yp11.32 - p11.2**	Mosaic 55%	**7,8 Mb**	Mosaic 50%	**10,3 Mb**
GAIN	**Yq11.21 - q11.223**	Mosaic 50%	**45,2 Mb**	Mosaic 65%	**10,3 Mb**
**Common CNA**		**GP27_TC**		**GP27_PBZ**	
GAIN	**1 q32.1**	No mosaic	**1,1 Mb**	No Mosaic	**1,1 Mb**
GAIN	**7 p22.3 - p11.2**	Mosaic 45%	**55,6 Mb**	Mosaic 71%	**55,4 Mb**
GAIN	**7 p11.2**	No Mosaic	**0,35 Mb**	No Mosaic	**0,895 Mb**
GAIN	**7 q11.21 - q21.3**	Mosaic 46%	**32,1 Mb**	Mosaic 56%	**37,7 Mb**
LOSS	**7 q31.1**	Mosaic 28%	**2,3 Mb**	Mosaic 62%	**2,2 Mb**
GAIN	**7 q31.1 - q31.31**	Mosaic 46%	**7,9 Mb**	Mosaic 61%	**5,5 Mb**
LOSS	**9 p24.3 - p13.1**	Mosaic 26%	**38,8 Mb**	Mosaic 63%	**38,9 Mb**
LOSS	**9 p21.3**	Mosaic 71%	**0,863 Mb**	No Mosaic	**1,5 Mb**
LOSS	**10 p13 - p11.21**	Mosaic 36%	**21,8 Mb**	Mosaic 66%	**21,6 Mb**
LOSS	**10 q11.21 - q26.3**	Mosaic 35%	**93 Mb**	Mosaic 62%	**93 Mb**
GAIN	**12 q13.3 - q14.1**	No mosaic	**0,31 Mb**	No mosaic	**0,31 Mb**
LOSS	**15 q14 - q21.3**	Mosaic 37%	**17,9 Mb**	Mosaic 67%	**17,8 Mb**
LOSS	**16 p13.11 - p12.1**	Mosaic 46%	**10,3 Mb**	Mosaic 66%	**10,5 Mb**
GAIN	**19 p13.3 - p12**	Mosaic 41%	**23,4 Mb**	Mosaic 41%	**23,8 Mb**
GAIN	**19 q12 - q13.43**	Mosaic 37%	**30,5 Mb**	Mosaic 47%	**30,5 Mb**
GAIN	**20 p13 - p11.1**	Mosaic 38%	**26 Mb**	Mosaic 67%	**25,9 Mb**
GAIN	**20 q11.21 - q13.33**	Mosaic 37%	**32,9 Mb**	Mosaic 62%	**32,5 Mb**

Table S3. CNAs identified in PBZs. Orange colour: GAIN exclusive of a PBZ in a specific patient, but present in other samples (TC or GSC) of other patients; green colour: LOSS exclusive of a PBZ in a specific patient, but present in other samples (TC or GSC) of other patients; yellow colour: CNA exclusive and unique of PBZ; white colour: CNA identified in PBZ but also in other samples of the same patient and in others.

Locus	Genes	CNA	PBZ	Note
1 p36.33 - p36.32 2.003.279 -3.520.911	PRKCZ, PRKCZ-AS1, FAAP20, SKI, MORN1, LOC100129534, RER1, PEX10, PLCH2, LOC100996583, PANK4, HES5, TNFRSF14, TNFRSF14-AS1, PRXL2B, MMEL1, TTC34, ACTRT2, PRDM16-DT, PRDM16, MIR4251, ARHGEF16, MEGF6, MIR551A	GAIN	20	It is present in TC10, 12, 18 and 23 in loss; in GSC6, 10 and 12 in loss.
1p34.2 41.355.091 - 41.479.154	SCMH1	GAIN	10	
2p24.3 14.643.522- 16.273.174	LRATD1, NBAS, DDX1, LINC01804, MYCNUT, MYCNOS, MYCN, GACAT3	GAIN	22	It is present in GSC22 and 7 in loss; in GSC22 and 8 in gain.
2p23.3 27.177.631- 27.509.817	CGREF1, ABHD1, PREB, PRR30, TCF23, SLC5A6, ATRAID, CAD, SLC30A3, DNAJC5G, TRIM54, UCN, MPV17, GTF3C2, GTF3C2-AS1, EIF2B4, SNX17, ZNF513, PPM1G, FTH1P3, NRBP1	GAIN	15	It is present in TC22 in gain; in GSC22 in loss; in GSC8 in gain.
2q14.2 121.316.763 - 121.401.255	GLI2	GAIN	27	
3q13.31 117.895.555- 118.070.129	TUSC7, MIR4447	GAIN	22	It is presenti in TC15 in gain.
3q26.33 181.075.904- 181.216.981	PEX5L, PEX5L-AS2	GAIN	15	It is present in TC5 and 22 in gain; in GSC8 in gain.
4q22.3 95.591.685- 95.664.017	PDLIM5	GAIN	22	It is present in TC15 in gain.
4q28.3-q31.1 139.437.734- 139.651.195	LINC00499	GAIN	22	It is present in TC15 in gain; in GSC24 in gain; in GSC21 in loss.
5p15.33 204.737- 2.855.425	PLEKHG4B, LRRC14B, CCDC127, SDHA, HRAT5, PDCD6, AHRR, EXOC3-AS1, EXOC3, SLC9A3, PP7080, SLC9A3-AS1, MIR4456, LOC100996325, CEP72, TPPP, ZDHHC11B, ZDHHC11, BRD9, TRIP13, LOC100506688, NKD2, SLC12A7, MIR4635, CTD-3080P12.3, SLC6A19, SLC6A18, TERT, MIR4457, CLPTM1L, LINC01511, SLC6A3, LPCAT1, MIR6075, SDHAP3, LOC728613, MIR4277, MRPL36, NDUFS6, LINC02116, IRX4, CTD-2194D22.4, LOC100506858, LSINCT5, IRX2, C5orf38	LOSS	10	It is present in TC5, 8, 14,18 and 21 in loss; in GSC17 and 22 in gain; in GSC21 in loss.
5q13.1 - q13.2 68.169.205 - 71.645.716	LINC02198, SLC30A5, SNORA50D, CCNB1, CENPH, MRPS36, CDK7, CCDC125, AK6, TAF9, RAD17, SMA4, MARVELD2, LOC101928924, OCLN, OCLNP1, SNORD13B-2, SNORD13B-1, GTF2H2C_2, GTF2H2B, GTF2H2, GTF2H2C, GUSBP3, LOC653080, GUSBP9, LOC101929599, LOC102724995, SERF1A, SERF1B, SMN2, SMN1, LINC02197, SMA5, LOC441081, NAIP, PMCHL2, BDP1, MCCC2, CARTPT, MAP1B, MIR4803, MRPS27	LOSS	24	It is present in GSC17 and 22 in gain; in GSC8 in loss.
5q13.3 74.546.402 - 74.669.810	ANKRD31, HMGCR	GAIN	22	It is present in TC15 in gain; in GSC17 and 22 in gain, in GSC8 in loss.
6q16.3 101.976.316 - 103.286.627	GRIK2	GAIN	10	It is present in TC5, 7, 9 and 26 in loss;in GSC7 and 26 in loss.
6q21 105.907.606 - 105.952.440	PREP	GAIN	22	It is present in TC27 in gain; in TC5, 7, 9 and 26 in loss; in GSC7 and 26 in loss.
7q21.12 86.232.435- 86.412.277	GRM3, KIAA1324L	GAIN	22	It is present in TC7, 9, 11, 12, 13, 14, 18, 20, 23, 24, 26 and 27 in gain; in GSC7, 12, 13, 17, 18, 20, 22, 23 and 26 in gain.
7q36.1 148.879.636- 148.961.160	ZNF767P	GAIN	22	It is present in TC7, 9, 11, 12, 13, 14 15, 18, 20, 23, 24, and 26 in gain; in GSC6, 7, 12, 13, 17, 18, 20, 22, 23 and 26 in gain.

8q24.3 142.274.933-145.836.174	DENND3, SLC45A4, LOC105375787, LINC01300, GPR20, PTP4A3, MROH5, MIR1302-7, MIR4472-1, LINC00051, TSNARE1, ADGRB1, ARC, LOC101928087, JRK, PSCA, LY6K, LNCOC1, THEM6, SLURP1, LYPD2, SLURP2, LYNX1-SLURP2, LYNX1, LY6D, GML, CYP11B1, CYP11B2, LY6E-DT, CDC42P3, LY6E, C8orf31, LY6L, LY6H, GPIHBP1, ZFP41, GLI4, MINCR, ZNF696, TOP1MT, RHPN1-AS1, RHPN1, MAFA-AS1, MAFA, ZC3H3, SNORD149, GSDMD, LOC100310756, MROH6, NAPRT, EEF1D, TIGD5, PYCR3, TSTA3, ZNF623, ZNF707, BREA2, CCDC166, LOC101928160, MAPK15, FAM83H, MIR4664, FAM83H-AS1, LOC105375800, SCRIB, MIR937, PUF60, NRBP2, MIR6845, EPPK1, PLEC, MIR661, PARP10, GRINA, SPATC1, SMPD5, OPLAH, MIR6846, EXOSC4, MIR6847, GPAA1, CYC1, SHARPIN, MAF1, WDR97, HGH1, MROH1, MIR7112, SCX, BOP1, HSF1, DGAT1, MIR6848, SCRT1, TMEM249, FBXL6, SLC52A2, LOC101928902, ADCK5, CPSF1, MIR939, MIR6849, SLC39A4, VPS28, TONSL, TONSL-AS1, MIR6893, CYHR1, MIR10400, KIFC2, FOXH1, PPP1R16A, GPT, MFSD3, RECQL4, LRRC14, LRRC24, C8orf82, ARHGAP39	GAIN	24	It is present in TC20 in gain; in TC6 and 18 in loss; in GSC6 and 8 in loss.
9q13 - q34.3 70.341.555-140.073.968	MORE THAN 700 GENES	LOSS	27	It is present in TC8 in gain; in TC5 and 27 in loss; in GSC6 in loss.
9q34.2 - q34.3 134.962.968-139.498.580	MORE THAN 550 GENES	GAIN	24	It is present in TC8 and 20 in gain; in TC18 and 27 in loss; in GSC6 in loss.
10p15.1 2.683.007- 4.386.320	LOC101927824, PFKP, SNORD142, PITRM1, PITRM1-AS1, LOC105376360, LINC02669, KLF6, LINC02639, MIR6078, LOC101927964, LINC00702	LOSS	10	It is present in TC26 in gain; in TC5, 7, 9, 11, 12, 13, 14, 18, 23 and 24 in loss; in GSC26 in gain; in GSC6, 7, 12, 13, 18, 20, 22, 23 and 24 in loss.
10q21.3 69.866.933 - 70.674.274	DNA2, SLC25A16, TET1, CCAR1, SNORD98, STOX1, DDX50, DDX21, KIF1BP, SRGN, VPS26A, SUPV3L1, LOC101928994, HKDC1	LOSS	22	It is present in TC5, 7, 8, 9, 11, 12, 13, 14, 18, 23, 24, 26 and 27 in loss; in GSC6, 7, 12, 13, 18, 20, 22, 23, 24 and 26 in loss.
10q26.13 125.111.028-126.835.056	GPR26, CPXM2, CHST15, OAT, NKX1-2, LHPP, FAM53B, FAM53B-AS1, EEF1AKMT2, ABRAXAS2, ZRANB1, CTBP2, MIR4296	LOSS	27	It is present in TC5, 7, 8, 9, 11, 12, 13, 14, 18, 23, 24, 26 and 27 in loss; in GSC6, 7, 8, 12, 13, 18, 20, 22, 23, 24 and 26 in loss.
10q26.3 134.767.642 - 135.026.325	ADGRA1, KNDC1, UTF1, VENTX, MIR202HG, MIR202, ADAM8, TUBGCP2, ZNF511, CALY, PRAP1, FUOM, ECHS1	LOSS	10	It is present in TC5, 6, 7, 8, 9, 11, 12, 13, 14, 18, 23, 24, 26 and 27 in loss; in GSC6, 7, 8, 12, 13, 18, 20, 22, 23, 24 and 26 in loss.
11p11.2 44.086.101-44.149.848	EXT2	GAIN	9 and 22	It is present in TC11, 15 in gain; in TC18 in loss; in GSC6 and 26 in gain.
11q22.3 108.441.303-109.335.335	C11orf87	LOSS	27	It is present in TC11 in gain; in GSC6 in loss.
12p13.31 7.696.275- 8.102.210	APOBEC1, GDF3, DPPA3, CLEC4C, NANOGNB, NANOG, SLC2A14, SLC2A3, FOXJ2, C3AR1	LOSS	27	It is present in TC21 in gain; in TC18 and 26 in loss; in GSC26 in loss.
12q14.1 56.405.238-56.516.952	AGAP2, AGAP2-AS1, TSPAN31, CDK4, MIR6759, MARCHF9, CYP27B1, METTL1, EEF1AKMT3, TSFM, AVIL, CTDSP2, MIR26A2	GAIN	22	It is present in TC5, 10, 13, 24 and 27 in gain; in TC13, 21 and 24 in loss; in GSC8, 10, 13, 22 and 24 in gain; in GSC8, 13 and 22 in loss.

13q21.32 65.899.949-66.375.742	PCDH9, PCDH9-AS2	LOSS	27	It is present in TC8, 13, 23 and 24 in loss; in GSC6, 8 and 13 in loss.
13q21.1 56.646.284-57.104.133	PCDH17	GAIN	15	It is present in TC8, 13, 23 and 24 in loss; in GSC6, 8 and 13 in loss.
14q11.2 - q12 22.545.671-24.112.628	C14orf93, PSMB5, PSMB11, CDH24, ACIN1, C14orf119, LMLN2, CEBPE, SLC7A8, RNF212B, HOMEZ, PPP1R3E, BCL2L2, BCL2L2-PABPN1, PABPN1, SLC22A17, EFS, IL25, CMTM5, MYH6, MIR208A, MYH7, MHRT, MIR208B, NGDN, THTPA, ZFHX2, AP1G2, LOC102724814, JPH4, DHRS2, DHRS4-AS1, DHRS4, DHRS4L2, DHRS4L1, CARMIL3, CPNE6, NRL, PCK2, DCAF11, FITM1, PSME1, EMC9, PSME2, MIR7703, RNF31, IRF9, REC8, IPO4, TM9SF1, TSSK4, CHMP4A, MDP1, NEDD8-MDP1, NEDD8, GMPR2, TINF2, TGM1, RABGGTA, DHRS1, NOP9, CIDEB, LTB4R2, LTB4R, ADCY4, RIPK3, NFATC4, NYNRIN, CBLN3, KHNYN, SDR39U1, LOC101927045, CMA1, CTSG	GAIN	22	It is present in TC5, 7 and 18 in loss; in GSC7 in loss.
16p13.3 1.040.696- 1.816.078	SSTR5-AS1, SSTR5, C1QTNF8, CACNA1H, TPSG1, TPSB2, TPSAB1, TPSD1, UBE2I, BAIAP3, TSR3, GNPTG, UNKL, C16orf91, PERCC1, CCDC154, CLCN7, PTX4, TELO2, IFT140, TMEM204, LOC105371046, CRAMP1, JPT2, MAPK8IP3, MIR3177, NME3, MRPS34, EME2, SPSB3, NUBP2, IGFALS, HAGH	GAIN	15 and 27	It is present in TC20 in gain; in TC6, 18, 23 and 24 in loss; in GSC26 in gain; in GSC6 and 23 in loss.
16q13 56.062.663-56.340.911	POLR2C, DOK4, CCDC102A, ADGRG5, ADGRG1, ADGRG3, DRC7, KATNB1	GAIN	24	It is present in TC7, 10, 15, 17, 22 and 23 in gain; in TC6, 18, 24 in loss; in GSC6 and 23 in gain; in GSC6 in loss.
16q24.2 - q24.3 87.034.408 - 87.891.103	ZNF469, ZFPM1, MIR5189, LOC100128882, ZC3H18-AS1, ZC3H18, IL17C, CYBA, MVD, SNAI3-AS1, SNAI3, RNF166, CTU2, PIEZO1, MIR4722, LOC100289580, LOC339059, CDT1, APRT, GALNS, TRAPPC2L, PABPN1L, CBFA2T3, LOC101927793, LOC100129697, ACSF3, LINC00304, LINC02138, CDH15, SLC22A31, ZNF778, ANKRD11, LOC105371414	LOSS	27	It is present in TC20 in gain; in TC5, 7, 18 and 24 in loss; in GSC26 in gain; in GSC6 and 7 in loss.
17q22 53.976.570 - 54.618.904	SEPTIN4-AS1, SEPTIN4, TEX14, RAD51C, PPM1E, TRIM37, SKA2, MIR454, MIR301A, PRR11	LOSS	24	It is present in TC10 in gain: in TC18 and 23 in loss; in GSC10 and 17 in gain; in GSC17 and 23 in loss.
18p11.31 5.226.858 -5.382.924	LINC00526, LINC00667, ZBTB14, EPB41L3	GAIN	22	It is present in TC5 in gain; in GSC6 and 17 in loss.
18p11.21 13.230.838-13.353.624	LDLRAD4, C18orf15	GAIN	15	It is present in TC22 in gain; in GSC6 in gain; in GSC17 in loss.
22q12.3 34.638.273-34.741.628	RBFOX2	GAIN	15	in TC5, 9, 10, 11, 18 and 20 in loss; in GSC24 in gain; GSC10 in loss.
22q12.3 35.602.505 - 35.664.769	NCF4, CSF2RB	GAIN	22	It is present in TC15 in gain; in TC5, 9, 10, 11, 18 and 20 in loss; in GSC24 in gain; GSC10 and 20 in loss.

Table S4. CNAs shared in PBZs and in GSCs. For each imbalance is reported the type of alteration (loss or gain), the lenght (in Mb) and the percentage of mosaicism. Yellow colour identified CNAs exclusive of PBZs and GSCs.

Common CNA		PBZ10		GSC10	
LOSS	**2p22.3 - p21**	33%	**11,2 Mb**	No mosaic	**11,6 Mb**
GAIN	**4q12 - q13.1**	No mosaic	**8,1 Mb**	No mosaic	**8,2 Mb**
LOSS	**8p23.2 - p12**	38%	**31,7 Mb**	No mosaic	**31,7 Mb**
LOSS	**8p12**	34%	**2,3 Mb**	No mosaic	**0,725 Mb**
LOSS	**8p11.23**	34%	**2,3 Mb**	No mosaic	**38,7 Mb**
GAIN	**9p24.2 - p22.1**	No mosaic	**16,1 Mb**	No mosaic	**16,6 Mb**
LOSS	**9p21.3**	51%	**6,4 Mb**	No mosaic	**5 Mb**
GAIN	**9p21.1 - p13.3**	No mosaic	**2,5 Mb**	No mosaic	**2 Mb**
LOSS	**9p13.3 - p13.1**	39%	**4,2 Mb**	No mosaic	**4,8 Mb**
LOSS	**10p15.3**	38%	**4,1 Mb**	No mosaic	**2,5 Mb**
LOSS	**10q24.32 - q25.1**	46%	**4,2 Mb**	No mosaic	**5,5 Mb**
GAIN	**12q12 - q21.1**	58%	**35,3 Mb**	No mosaic	**35,2 Mb**
GAIN	**12q15 - q21.1**	No mosaic	**5,5 Mb**	No mosaic	**4,9 Mb**
GAIN	**12q21.31 - q21.32**	No mosaic	**1,6 Mb**	No mosaic	**1,4 Mb**
GAIN	**12q21.33 - q22**	No mosaic	**2,5 Mb**	No mosaic	**2,3 Mb**
LOSS	**12q22**	39%	**2,3 Mb**	No mosaic	**2,7 Mb**
GAIN	**12q22 - q23.1**	47%	**7,9 Mb**	No mosaic	**1,9 Mb**
GAIN	**12q23.1 - q23.3**	47%	**7,9 Mb**	No mosaic	**4 Mb**
LOSS	**12q24.32 - q24.33**	50%	**6,5 Mb**	No mosaic	**6,5 Mb**
LOSS	**14q23.1 - q31.1**	35%	**21,3 Mb**	No mosaic	**28,8 Mb**
LOSS	**20q13.33**	52%	**2,4 Mb**	No mosaic	**2,6 Mb**
LOSS	**22q11.21 - q13.33**	48%	**29,8 Mb**	No mosaic	**29,2 Mb**
**Common CNA**		**PBZ22**		**GSC22**	
GAIN	**2p24.3**	No mosaic	**1,6 Mb**	No mosaic	**1,5 Mb**
GAIN	**5p13.2**	No mosaic	**150 kb**	75%	**45,9 Mb**
GAIN	**5q13.3**	No mosaic	**2,1 Mb**	75%	**130,9 Mb**
GAIN	**7q21.12**	No mosaic	**179,8 kb**	No mosaic	**1,3 Mb**
LOSS	**10q21.3**	47%	**807 kb**	75%	**93 Mb**
GAIN	**12q14.1**	No mosaic	**111,7 kb**	No mosaic	**111,7 kb**
GAIN	**12q15**	No mosaic	**1,9 Mb**	No mosaic	**1,9 Mb**
**Common CNA**		**PBZ24**		**GSC24**	
LOSS	**12q13.13**	59%	**1,1 Mb**	No mosaic	**17,5 Mb**
GAIN	**12q13.3-q14.1**	No mosaic	**360 kb**	No mosaic	**1,5 Mb**
GAIN	**Yp11.31-p11.2**	50%	**10,2 Mb**	4%	**7,8 Mb**
GAIN	**Yq11.21-q11.223**	65%	**10,2 Mb**	50%	**44,9 Mb**
